# Pharmacotherapy of Schizophrenic Patients: Preponderance of Off-Label Drug Use

**DOI:** 10.1371/journal.pone.0003150

**Published:** 2008-09-10

**Authors:** David Pickar, Jessie Vinik, John J. Bartko

**Affiliations:** 1 Gabriel Sciences, LLC, Cabin John, Maryland, United States of America; 2 Department of Psychiatry, Johns Hopkins Medical School, Baltimore, Maryland, United States of America; 3 Department of Psychiatry, Uniformed Services University of the Health Sciences, Bethesda, Maryland, United States of America; 4 Department of Statistics, University of Maryland School of Medicine, Baltimore, Maryland, United States of America; James Cook University, Australia

## Abstract

Multiple drug class combinations are often prescribed for the treatment of schizophrenia, although antipsychotic monotherapy reflects FDA labeling and scientific justification for combinations is highly variable. This study was performed to gain current data regarding drug treatment of schizophrenia as practiced in the community and to assess the frequencies of off-label drug class combinations. 200 DSM IV-diagnosed schizophrenic patients recruited from community treatment sources participated in this cross-sectional study of community based schizophrenic patients. Drug class categories include First and Second Generation Antipsychotic drugs (FGA and SGA, respectively), mood stabilizers, antidepressants and anti-anxiety drugs. 25.5% of patients received antipsychotic monotherapy; 70% of patients received an antipsychotic and another drug class. A total of 42.5% of patients received more than one antipsychotic drug. The most common drug class combination was antipsychotic and a mood stabilizer. Stepwise linear discriminant function analysis identified the diagnosis of schizoaffective schizophrenia, history of having physically hurt someone and high scores on the General Portion of the PANSS rating scale predicted the combined use of an antipsychotic drug and a mood stabilizer. “Real world” pharmacotherapy of schizophrenia has developed its own established practice that is predominantly off-label and may have outstripped current data support. The economic implications for public sector payers are substantial as well as for the revenue of the pharmaceutical industry, whose promotion of off-label drug use is an increasingly problematic. These data are consistent with the recognition of the therapeutic limitations of both first and second generation antipsychotic drugs.

## Introduction

Off-label medication use, the clinical application of prescribed drugs for indications other than those evaluated and approved by the Food and Drug Administration (FDA), is widespread in many areas of medicine[1]. Although there is considerable literature related to the use of mood stabilizers, antidepressants and anti-anxiety drugs added to antipsychotic drug treatment [2]–[17], none of these combinations are approved by the FDA for the treatment of schizophrenia. While off-label uses are legal and in many instances may be in the best interest of patients, they have not received the same degree of independent scrutiny through randomized clinical trials as have indications approved by the FDA. Industry sponsors may be hesitant to submit an already approved drug for a new indication because of what may be perceived as unnecessary expense and the considerable risk of not meeting primary endpoints with randomized controlled trials. Radley and co-workers [1] examined off-label prescribing patterns of office based physicians, distinguishing treatments as having strong or limited scientific support and found that the greatest disparity between “supported and unsupported” off-label prescriptions occurred among psychiatric therapies (4% strong support vs 96% limited or no support).

Clozapine is unique among antipsychotic drugs as its indication specifies that clozapine is “indicated for the management of severely ill schizophrenic patients who fail to respond adequately to standard drug treatment for schizophrenia;” and “for reducing the risk of recurrent suicidal behavior in patients with schizophrenia or schizoaffective disorder who are judged to be at chronic risk for re-experiencing suicidal behavior, based on history and recent clinical state”[18]. The unique effectiveness of clozapine contributed to the early wave of optimism regarding the therapeutic superiority of other members of the so-called Second Generation Antipsychotic drugs (SGA's) [19] a notion supported in some measure by meta-analysis.[20] Results from the recent non-industry funded, multi-centered CATIE trial carried out in the United States [21] and CUtLASS1trial [22] carried out in the UK, however, have judiciously challenged the notion of superiority of SGA over First Generation Antipsychotic Drugs (FGAs) in the treatment of schizophrenia. In both trials, FGAs performed remarkably well in comparison to SGAs (clozapine not included) with regard to symptom reduction, side effect profile and cost utility [21]–[26]. Although these findings may have been unexpected, in actuality, these studies are in substantive agreement with FDA labeling: the effectiveness of SGAs (clozapine excluded) is no better than FGAs for the treatment of schizophrenia.

Given the severity of schizophrenia and the limitations of the effectiveness of antipsychotic drugs it is not surprising that clinicians have turned to numerous empirical approaches to enhance clinical response. We report here patterns of pharmacotherapy including drug class combinations used in the treatment of seriously ill, community based schizophrenic patients. Off-label treatments and the emerging community practice standards for the treatment of schizophrenia are identified.

## Methods

Two hundred outpatients participated in this study. Each patient provided written informed consent for participation in the protocol approved by Western Institutional Review Board (WIRB), Seattle Washington and received $75 for participation. All research procedures were carried out by Gabriel Pharma. WIRB approved patient recruitment notices were sent to community treatment settings, private clinicians and to the National Alliance for the Mentally Ill in the Washington DC and Montgomery County, MD area. Following patient self referral and initial screening by Gabriel Pharma, each patient met with a member of the Gabriel Pharma research team during which time the research protocol was explained and questions were encouraged. After providing written informed consent, he/she participated in a structured interview detailing psychiatric, medical and drug treatment histories and provided a venous drug collection for DNA analysis (data not reported here). A total of 200 patients participated in the protocol from August, 2004 through March 2006.. Participating patients responded to recruitment notices from the following: : St Luke's House, Inc (25%, a private, non-profit organization that offers integrated treatment and housing for the mentally ill in Montgomery County, MD; Anchor Mental Health (23.5%), Catholic Charities' full service community treatment center for the mentally ill in Washington, DC; Green Door (10.5%), a private non-profit Washington, DC community program dedicated to aiding patients with mental illness to return to work and live independently; National Alliance for the Mentally Ill (NAMI) (10.5%) the nation's largest grassroots organization for people with mental illness and their families; Washington Assessment and Therapy Services (WATS) (8%), a private non-profit behavioral health center in Gaithersburg, MD that provides services for the mentally ill; DC Department of Mental Health (4.5%), a Washington, DC government agency that provides comprehensive mental health services; NIH patients (9%) who had previously participated in schizophrenia treatment protocols; Woodley House (2%), a private non-profit program for the mentally ill that was the first community based residential program for the mentally ill in the United States; and Private Practice referrals (7%).

The PI (DP) administered PANSS [27] and Montgomery-Asberg rating scales [28]; DSM IV [29] diagnosis was made by consensus after reviewing results of the clinical interview process. All patients had a DSM IV Axis I diagnosis of schizophrenia (Table 1). The mean (SD) age of patients who participated in the study was 45.1 (9.6) years and age of onset of illness was 19.9 (8.9) of age. Nineteen per cent of patients had a BMI less than 24.9 (normal or underweight); 40% had a BME 25–29.9 (overweight); and 41% had a BMI 30 or greater (obese). Current medications at the time of evaluation were reported by each patient and confirmed with notation from referring clinicians/case manager and by medical records when available.

**Table 1:** Patient Demography.

		Count	Percent
**DSM IV DX**			
	295.1 - Disorganized	2	1
	295.3 - Paranoid	50	25
	295.6 - Residual	3	1.5
	295.7 - Schizoaffective	56	28
	295.9 Undifferentiated	89	44
**Gender**			
	Female	81	40.5
	Male	119	59.5
**Race** 1			
	African American	104	52.
	Asian	6	3
	Caucasian	90	45
**Residence**			
	Family Home	26	13
	Non Supervised Dwelling	60	30
	Shelter	5	2.5
	Supervised Dwelling	109	54.5
**Marital Status**			
	Divorced/Separated	22	11
	Married	18	9
	Never Married	150	75
	Other	10	5
**Involuntary Hospitalization/s**			
	No	105	52.5
	Yes	95	47.5
**Suicide Attempt/s**			
	No	104	52.0
	Yes	96	48.0
**Jail**			
	No	103	51.5
	Yes	97	48.5
**Ever Hurt Someone**			
	No	141	70.5
	Yes	59	29.50

1 Self-reported race classification per NIH Guidelines.

The classes of medications reported here were: antipsychotic drugs (FGAs and SGAs), mood stabilizers, anti-depressants and anti-anxiety agents. Medicationcombinations are reported as “exclusive” indicating that the combination is the sole treatment, or as “non-exclusive” in which case other drug classes might have also have been administered. All percentages are of the total patient population (200) unless otherwise noted.

Linear discriminant function was applied to demographic and rating variables shown in Tables 1 and 2 as independent variables to predict the two most common medication class combinations: antipsychotic and mood stabilizer; antipsychotic and antidepressant as noted in the text.

**Table 2:** Mean (SD) of Total Number of Hospitalizations and Rating Scale scores.

Total # of Hospitalizations	7.5 (7.4)
PANSS Total	110.9 (16.5)
PANSS Positive	25.8 (5.6)
PANSS Negative	28.3 (4.5)
PANSS General Psych	56.8 (4.5)
Montgomery-Asberg Total	31 (8.23)

## Results

Table 1 presents a summary of demographic and clinical variables and Table 2 presents the means of PANSS and Montgomery-Asberg Depression Rating Scale and the total number of hospitalizations. Table 3 presents the patterns of antipsychotic drug use and Table 4 details the frequencies of all possible medication combinations. “Non-exclusive” use of antipsychotic drugs and mood stabilizers would include the total frequency of antipsychotic and other medication classes.

**Table 3:** Antipsychotic Usage.

Medication Abbreviaton	Treatment	Count	Percent
*FGA = First Generation Antipsychotic Drug*	SGA without FGA	148	74
*SGA = Second Generation Antipsychotic Drug*	FGA without SGA	15	7.5
	FGA+SGA	28	14
	No Antipsychotic Drug	9	4.5
	TOTAL	200	100
	Percent of FGA administration that also received SGA administration	28 of 43	65
	More than one SGA	60	30
	More than one FGA	8	4
	More than one APS	85	42.5

**Table 4:** All Medication Class Combinations.

Medication Class	Treatment	Count	Percent
*AA = Anti-Anxiety*	No Medication	5	2.5
*AD = Antidepressant*	AA	0	0
*MS = Mood Stabilizer*	AD	3	1.5
*APS = Anti-Psychotic*	MS	0	0
	APS	51	25.5
	AD+AA	0	0
	MS+AA	0	0
	MS+AD	0	0
	MS+AD+AA	1	.5
	APS+AA	5	2.5
	APS+AD+AA	8	4
	APS+AD	38	19
	APS+MS	51	25.5
	APS+MS+AA	8	4
	APS+MS+AD	22	11
	APS+MS+AD+AA	8	4
	TOTAL	200	100

In total, antipsychotic drugs were administered to 95.5% of the patients. SGA administration was far more prevalent than FGA administration (88% vs. 21.5%, respectively), with the majority of patients receiving SGA without concomitant FGA (Table 3). In contrast, the majority of patients who received FGA also received an SGA. Thirty per cent of the patient population were administered more than one SGA. In total, 42.5% of patients were treated with more than one antipsychotic drug (Table 3).

25.5% of patients were treated with antipsychotics as their sole medication class (Table 4) and 70% were treated with an antipsychotic plus another medication class (4.5% of patients were antipsychotic free). More than two drug classes were used in 23.5% of patients. The most common drug class combination was antipsychotic with mood stabilizer (25.5% exclusive; 45% non-exclusive) followed by antipsychotic with antidepressant (19% exclusive; 38% non-exclusive) and finally, antipsychotic with antidepressant and anti-anxiety (2.5% exclusive; 14% non-exclusive).

Olanzapine and risperidone were each administered to 26% of patients; quetiapine was next most prevalent (20.5%) following by clozapine (18%), aripiprazole (14%), ziprasidone (11%), haloperidol (7%), depot injections (haloperidal+fluphenazine) (5%) and 1.5% other FGA's. Divalproex was the most common mood stabilizer (26% of patients) followed by lithium (5.5%), topiramate (5%), carbamazepine, gabapentin and lamatrogine each of which was administered to 2.5% of patients. Fluoxetine, buproprion and paroxetine were each administered to 7% of patients while venlafaxine and escitalopram were administered to 5% of patients and citalopram to 2.5% of patients. Clonazepam (6.5%) and lorazepam (3.5%) were the most frequently administered anti-anxiety agents.

Table 5 shows statistically significant results of stepwise discriminant linear function analyses in which the clinical and demographic variables were independent variables predicting antipsychotic plus mood stabilizer and antipsychotic plus antidepressant, the combinations with the largest frequencies. Antipsychotic plus mood stabilizer exposure was significantly predicted (67% correct classification, (p<0.001) by: 1) diagnosis of schizoaffective schizophrenia; 2) history of having hurt someone; and 3) high scores on the General Psych Portion of the PANSS Scale. Antipsychotic plus antidepressant use (67% correct classification, p<0.001) was predicted by greater number of hospitalizations and higher score on the Montgomery-Asberg depression rating scale.

**Table 5:** Results of Linear Discriminant Function.

Clinical Predictor	APS+MS n = 89	APS+AD n = 76
DX	F = 15.69, p<.001	
Total # hospitalizations		F = 7.48, p = .006
Ever Hurt Someone	F = 6.0, p = .015	
Montgomery-Asberg Total		F = 6.7, p = .01
PANNS Gen Psych	F = 5.34, p = .022	

## Discussion

The core finding from this study of seriously ill community based schizophrenic patients is the predominance (70%) of off-label medication administration with the use of antipsychotic as a sole medication class (per FDA label) a relatively infrequent occurrence (25.5%). Despite emerging data suggesting relative therapeutic equivalence, SGA's were far more frequently administered than were FGAs (88 vs 21.5%); when FGA administration occurred it was overwhelmingly as an addition to SGAs: 65% of FGA administration was with concomitant SGAs. This study's sample was clinically well characterized; demographics are is reflective of highly symptomatic patients with schizophrenia in midlife. For example, there was a slightly higher male prevalence (59.5%); patients were largely never married (75%); a majority live in supervised dwelling (54.5%); and nearly half had had involuntary hospitalizations (47.5%), had been in jail (48.5%), and had made a suicide attempt (48%); mean age of onset was 19.9 years. These demographies place the study population into the mainstream of community based seriously mentally is patients with schizophrenia The relatively high prevalence of clozapine administration (18%) is consistent with the high severity of illness of our population as reflected by their behavioral ratings.. The relatively low prevalence of the Schizophrenia, Disorganized Type is likely due to the evaluation of patients through the “window” of medication treatment rather than in the drug free state. 79% of the population was overweight or obese, reflecting one of the numerous elements of enhanced health risk that most of these patients face. The most frequent combination of drug classes was a mood stabilizer added to an antipsychotic drug, followed by more than one antipsychotic, antipsychotic and antidepressant and antipsychotic and anti-anxiety combinations. The most commonly used APS were olanzapine (26%), and risperidone and quetiepine (20.5%) with frequency of clozapine administration at 18%.

Buchanan et al [30] reported that 50% of 344 schizophrenic outpatients were treated with either antidepressants, anti-anxiety or mood stabilizers concomitant with antipsychotic drugs and that 17% of patients were treated with more than one adjunctive agent. Tapp and colleagues [5] investigated the utilization of more than 1 antipsychotic and found in a survey of a diagnostically diverse group of schizophrenic outpatients that 13% received an FGA added to SGA, a comparable figure to the 13.5% frequency we observed for this combination. Baseline medication use of the 1,493 patients of the CATIE study [31] revealed a high frequency of no antipsychotic medication (26%) and a low frequency of more than 1 antipsychotic (5%) and antipsychotic plus mood stabilizer (including lithium) (15%) in comparison to the prevalence of no antipsychotic (4.5%), patients treated with more than one antipsychotic (42.5%) and patients treated with antipsychotic plus mood stabilizer (44.5%) in a our cohort. Our cohort of community-based patients who were not participating in a prospective double-blind controlled study was likely considerably more ill than CATIE patients as reflected by mean total PANSS score: 111±16.5 in our cohort vs 75.7±17.6 for CATIE.[21] Interestingly, baseline CATIE antipsychotic plus antidepressant (31%) and antipsychotic plus anti-anxiety (18%) treatment combinations 31) were comparable to prevalence among our patients (38% and 14.5%, respectively).

The predominance of off-label drug combinations speaks to the overriding message of CATIE [21] and CUtLASS 1 [22]: there are significant limitations in ineffectiveness of all antipsychotic drugs. There are, however, no clear standards or guidelines for the use of off-label treatments. Our multivariate model predicting use of antipsychotic drugs and concomitant mood stabilizers identified the diagnosis of schizoaffective schizophrenia, history of having hurt someone and high scores on the General Psychopathology subscale of the PANSS (which includes items such as “uncooperativeness,” “lack of judgment and insight,” “poor impulse control,”) as predictors. This suggests combined antipsychotic and mood stabilizers are used in patients with aggressive elements to their behavior. In contrast, greater number of hospitalizations and high Montgomery-Asberg depression ratings were predictors of concomitant antidepressant use, suggesting this approach in depressed schizophrenics [32] with high risk of relapse. We are unaware of data elsewhere related to clinical predictors of off label drug administration.

There are two critical elements to off-label prescribing practices related to the Food and Drug Administration: a drug approved for marketing may be labeled, promoted and advertised by the manufacturer for only those uses for which the drug's safety and effectiveness have been established by the FDA.[33]–[34] Industry practices regarding promotion of uses not included in the drug label have become increasingly scrutinized, as exemplified by the attention and penalty to market practices that encourage off-label use of the anticonvulsant, gabapentin.[35]–[36] The FDA has recently proposed new guidelines that enable sponsors to distribute publications about unapproved uses of approved drugs and advices. [37] Of serious concern, however, is that the selective use of peer-reviewed literature may not be able to satisfactorily ensure the quality of off-label promotion [38]–[40], contributing to the problematic oversight of industry's promotional efforts. In contrast to industry whose “behavior” in the marketplace is at least theoretically closely scrutinized, the clinician has considerable flexibility: if a product has been FDA approved, a physician may choose to prescribe it for uses or in treatment approaches or patient populations other than the approved indication[33]–[34]. It is the responsibility of the manufacturer to gain FDA approval for adding new uses to the product label. It is hardly surprising that a company may be hesitant or even resistant to invest the resources and entertain the risk of unfavorable results involved in FDA review of a new indication, given the multibillion dollar revenues for medications whose off label use in schizophrenia is described in this report Moreover, the impact of off label use in schizophrenia is particularly great on the public sector as schizophrenic patients' care is largely supported by Medicaid and to a lesser degree Medicaire. In a recent Wall Street Journal/Harris poll [41], the public appears evenly divided on whether physicians should (45%) or should not (46%) be allowed to prescribe medications for off-label uses; in contrast, a majority (62%) of respondents believe that pharmaceutical companies should not be allowed to encourage off-label use.

The scientific merits underlying the use of these off-label drug class combinations are variable; although it is an area where clinicians play a major role in the development of drug treatment[42] Radley et al [1] used the DRUGDEX [43] system, a highly recognized scientific documentation resource, to categorize off-label uses as having strong scientific support, limited scientific support or no scientific support. Their findings that 96% of psychiatric off-label uses have limited or no support might well be questioned by the psychiatric research community. The need for systematic evaluation of treatment efficacy of drug class combinations is clearly needed.

In summary, there is a predominance of off-label prescription use in the treatment of seriously ill patients with schizophrenia in the community. It appears that the real world pharmacotherapy of schizophrenia has developed its own established practice that may have outstripped current data support. The economic implications of off label use in schizophrenia for public sector payers as well as for the pharmaceutical industry are substantial. The independent research community could make an important contribution by supporting a program of systematic evaluation. What might such an undertaking look like from the perspective of clinical trial design? One clear and logical approach is to study the superiority, on some primary endpoint (e.g. total PANSS score) when the drug in question is added to an antipsychotic in comparison with antipsychotic monotherapy. In light of the very high drop out rate of the ambitious CATIE study, a design that enabled a high rate of subject completion (CATIE completion rate: 26%) would certainly be necessary for the study to have the necessary impact. It's unlikely that such work will stem from industry sponsored initiatives.
